# Population Expanding with the Phalanx Model and Lineages Split by Environmental Heterogeneity: A Case Study of *Primula obconica* in Subtropical China

**DOI:** 10.1371/journal.pone.0041315

**Published:** 2012-09-19

**Authors:** Hai-Fei Yan, Cai-Yun Zhang, Feng-Ying Wang, Chi-Ming Hu, Xue-Jun Ge, Gang Hao

**Affiliations:** 1 Key Laboratory of Plant Resources Conservation and Sustainable Utilization, South China Botanical Garden, Chinese Academy of Sciences, Guangzhou, China; 2 College of Life Sciences, South China Agricultural University, Guangzhou, China; 3 Shanghai Chenshan Plant Science Research Center, Chinese Academy of Sciences, Shanghai Chenshan Botanical Garden, Shanghai, China; 4 Graduate University of the Chinese Academy of Sciences, Beijing, China; George Washington University, United States of America

## Abstract

**Background:**

Current and historical events have both affected the current distribution patterns and intraspecific divergence of plants. While numerous studies have focused on the Qinghai-Tibetan Plateau (QTP), the impacts of such events on the flora of subtropical China remain poorly understood. Subtropical China is famous for its highly complex topography and the limited impact from glaciation during the Pleistocene; this may have resulted in a different genetic legacy for species in this region compared to fully glaciated areas.

**Methodology/Principal Findings:**

We used plastid and nuclear DNA sequence data and distribution modeling to analyze the divergence patterns and demographic history of *Primula obconica* Hance, a widespread herbaceous montane species in subtropical China. The phylogenetic analysis revealed two major lineages (lineage A and lineage B), representing a west-east split into the Yunnan and Eastern groups, and the Sichuan and Central groups, respectively. The Eastern and Central groups comprised relatively new derived haplotypes. Nested Clade Analysis and Bayesian Skyline Plot analyses both indicated that *P. obconica* mainly experienced a gradual expansion of populations. In addition, the simulated distribution of *P. obconica* during the Last Glacial Maximum was slightly larger than its present-day distribution.

**Conclusion/Significance:**

Our results are the first to identify a west-east migration of *P. obconica*. The gradual expansion pattern and a larger potential distribution range in cold periods detected for *P. obconica* indicate that the population expansion of this species is consistent with the phalanx model. In addition, the current patterns of genetic differentiation have persisted as a result of the extensive environmental heterogeneity that exists in subtropical China.

## Introduction

The distribution patterns and evolution of plants are profoundly impacted by their life history traits, environmental heterogeneity and historical events. However, the importance of these factors in the genetic divergence within and between species, along with the importance of changes in distribution patterns, remains a contentious issue [1]. There are two population expansion models reflecting the genetic legacy of the climatic oscillations and environmental heterogeneity. The pioneer model, first described by Hewitt [2], predicts that the pioneer (or edge) population expanding from a refugium will have relatively low genetic diversity as a result of founder effects. For example, the southern regions of Europe and North America are inhabited by a much greater number of species with more numerous subspecific divisions and greater allelic diversity than their northern counterparts [3]–[4], since vast areas of northern Europe and North America were repeatedly covered by massive ice-sheets during past glaciations [5]. In contrast, the phalanx model, first documented for alpine species [3], [6]–[7], describes the effects of slower expansions from refugia, with less significant bottlenecks than those encountered in pioneer-type expansions because many alleles are able to colonize sites over short distances [2]. Compared with the pioneer model which has been well documented in Europe and North America, the phalanx model has received comparatively little attention despite its potential importance in the evolution and demographic history of montane species.

Previous research on population expansions in China has mainly focused on the Qinhai-Tibetan Plateau (QTP), including the Himalaya-Hengduan Mountains (HHM). These studies indicated that the colonization patterns of most species in this region are consistent with the pioneer model, with populations on the QTP exhibiting relatively low genetic diversity and/or pure genetic haplotypes while those in the HHM exhibit high diversity [8]–[12]. However, the historical evolutionary relationships between species inhabiting the HHM and the adjacent eastern area (especially subtropical China) remain unknown.

**Figure 1:**
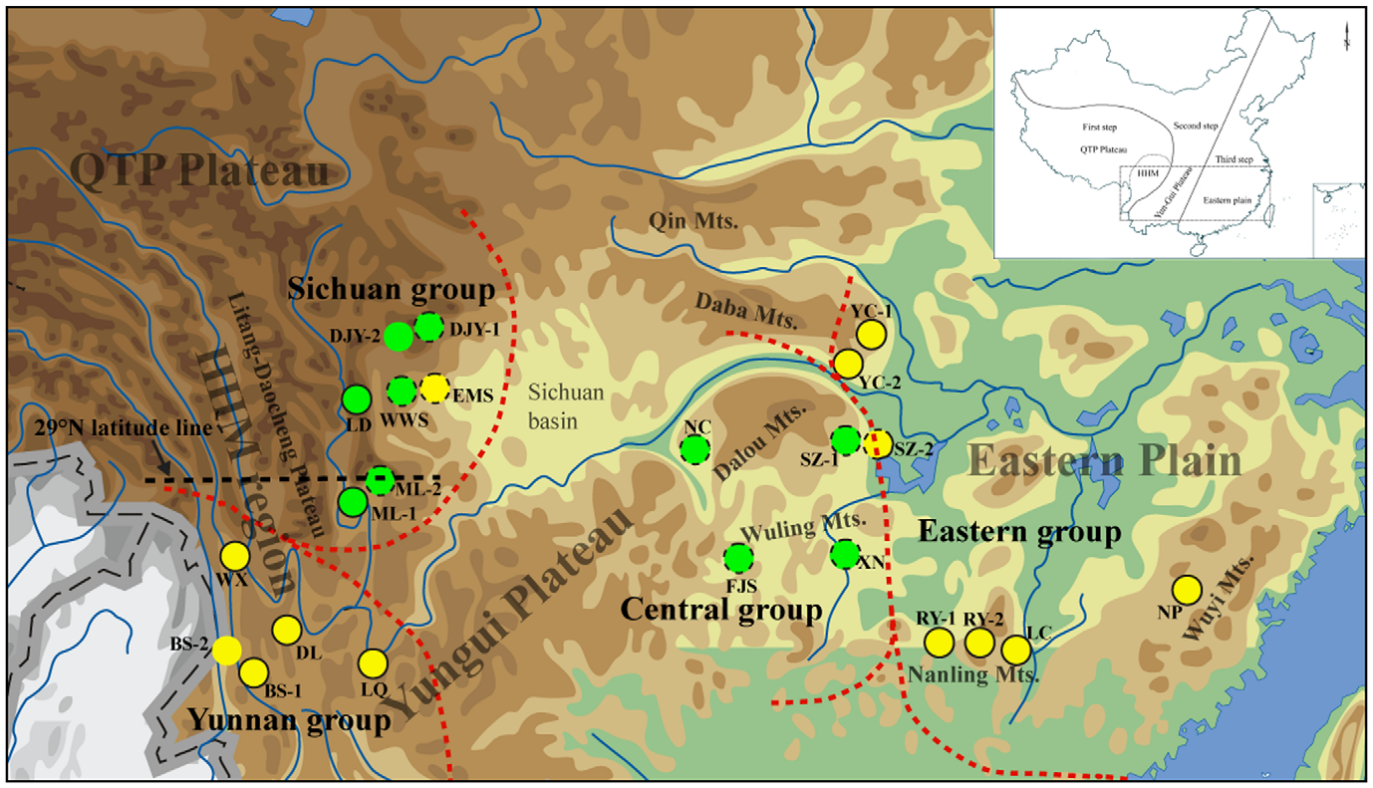
Sampling locations for the 23 populations of *Primula obconica* examined in this study. Circles with yellow and green colors correspond to lineages A and B (based on the plastid DNA dataset), respectively. Red dashed lines denote the lineage divergences identified by TCS and phylogenetic analyses, and the black dashed line indicated the 29°N latitudinal line in the HHM. The outer circle patterns (dashed or solid) correspond to the results from the ITS dataset. The inset map indicates the geographical features of China and the distribution range examined in the present study.

Subtropical China is famous for its complex topography. There is a remarkable decrease in altitude from west to east, which divided China into three geographic zones: the QTP, with an average elevation of 4000–5000 m a.s.l.; the eastern plain, which lies below 1000 m a.s.l.; and a transitional belt between the QTP and the eastern Plain ranging from 1000–2000 m a.s.l [13] (See inset map in Figure 1). Subtropical China spans all three of these zones: the HHM and the Yungui Plateau are examples of the first two, while the eastern plain is representative of the third. Additionally, there are also differences in the orientation of the mountain ranges in subtropical China. For example, many mountain ranges and large river systems that cross the HHM region run from north to south, while the Yungui Plateau and the eastern plain are oriented from west to east [13] (Figure 1). The complex topography of subtropical China provides varied micro-habitats for living organisms, which was used to explain its extremely high biodiversity compared to other areas in the region [14]–[16].

Subtropical China itself is thought to have been one of the most important refugia during the middle Miocene extinction (ca. 15 Mya) [17]. However, the impact of climatic oscillation during the Pleistocene on the present-day distribution of species is still being debated. One opinion, based on palaeovegetation data, is that evergreen broad-leaved forests in the subtropical region retreated southward to their present tropical zone during the Last Glacial Maximum (LGM) [18]–[20]. It is notable, however, that the subtropical region was never covered by massive ice sheets in the Pleistocene [21]. Consequently, this region might have harbored multiple refugia due to its mosaic of mountains and relatively mild environment [22], which are partially supported by resent studies [23]–[27].

As represented above, the population expansion of montane species in subtropical China might have occurred as described by the phalanx model, with species migrating down along mountain slopes and gradually expanding during cold periods but being forced to retreat upwards and form isolated island-like populations during the warm and moist interglacial periods. If this were the case, we would expect to detect a gradual expansion in effective population size, and a simulated species distribution in cold periods that is larger than at present.

The genus *Primula* exhibits the typical Chinese radiation pattern from the southwest to southeast [28]; the majority of *Primula* species in China are restricted to the southwest [29]–[30]. *Primula obconica* is a unique *Primula* species that has a relatively wide distribution range from southwest China (Yunnan and Sichuan provinces) to east China (Fujian province) stretching from 99° to 118°E and from 25° to 31°N, which means it is distributed in subtropical China [14], [29]. It mainly grows in moist thickets and deciduous forests at elevations of 500 to 3000 m [29], and its broad range suggests substantial adaptations to different environmental conditions. Six subspecies were recognized by morphology, viz., ssp. *obconica*, ssp. *begoniiformis* (Petitm.) W. W. Smith & Forr.; ssp. *parva* (I. B. Balfour) W. W. Smith & Forr.; ssp. *werringtonensis* (Forr.) W. W. Smith & Forr.; ssp. *nigroglandulosa* (W. W. Smith & Fletcher) C. M. Hu; and ssp. *fujianensis* C. M. Hu & G. S. He [29]–[31]. Of these subspecies, only ssp. *obconica* is distributed throughout almost the entire range. The other subspecies are all endemic and sympatric with ssp. *obconica* with the exception of the most recently described subspecies ssp. *fujianensis*, which occurs solely in the easternmost Fujian province [30]–[31]. The wild *P. obconica* was formally reported as diploid [32], although autotetraploid form was recorded as well [33]–[34]. Dowrick [33] pointed out that homostyly have not been found in the diploids but are present in the tetraploids, which means homostyly is significantly correlated with the polyploidy in *Primula* [35]–[36]. In our samplings, all wild populations were heterostylous except ssp. *fujianensis*, which is isolated both in distribution and reproduction from other subspecies of *P. obconica* [31].

In the present study, we used *P. obconica* as a model to test competing hypotheses to explain the migration and evolution of species in subtropical China by examining two polymorphic plastid loci and the complete nuclear ribosomal Internal Transcribed Spacer (ITS) region. Specifically, we aimed to determine: (i) whether the species originated from southwest China (or HHM), then eastward migrated; (ii) whether the phalanx or the pioneer model better describe the species' population expansion and demographic history; and (iii) the impact of environmental heterogeneity on the species' present-day distribution.

## Materials and Methods

### Ethics statement

No specific permits were required for the described field studies, since all samples were collected by researchers following current Chinese regulations. *Primula obconica* is not endangered nor protected in the sampled area, and none of the sampled locations are privately owned or protected by any law.

### Population sampling, DNA extraction, PCR and sequencing

Twenty-three populations were sampled throughout the distribution range of *P. obconica*, representing four subspecies (Figure 1, Table 1). *Primula obconica* ssp. *parva* and ssp. *nigroglandulosa* could not be sampled in this study because of their extremely limited distribution ranges [29]. Fresh leaves were immediately preserved in silica gel in the field, and total genomic DNA was extracted using the modified cetyltrimethyl ammonium bromide (CTAB) protocol [37]. Two plastid DNA regions (*trn*L*-trn*F and *rps*16) and a nuclear marker (ITS) were amplified [38]–[40]. Amplicons were purified using a DNA gel purification kit (TaKaRa) and sequenced in both directions using an ABI Big Dye Terminator Cycle-sequencing ready-reaction kit (Applied Biosystems) on an ABI 3700 DNA Analysis System (Applied Biosystems). An additional internal sequencing primer was designed to obtain the entire sequence of the *rps*16 region (hc-p1: 5′-GGTATGTTGCTGCCATTTTG-3′) due to homopolymer runs. All bidirectional sequencing reactions were carried out by Invitrogen Trading Shanghai Co., Ltd.

**Table 1:** Plant material, their sources, the detected chlorotypes and the genetic diversity identified in this study.

Nos.	Population codes	Locations	Longitude (E), Latitude (N)	Taxon	N	π (sd)	*H*_d_ (sd)	Chlorotypes (nos. of individuals)
	Lineage A: Eastern group				58	0.00122 (0.000122)	0.829	
1	NP	Nanping, Fujian	118°12′48″, 26°43′18″	ssp. *fujianensis*	6	0	0	C14(6)
2	LC	Lechang, Guangdong	113°00′17″, 25°01′00″	ssp. *obconica*	9	0.00023 (0.00010)	0.389 (0.164)	C3(2), C13(7)
3	RY-1	Ruyang, Guangdong	113°03′42″, 24°51′19″	ssp. *obconica*	8	0.00119 (0.00065)	0.464 (0.200)	C4(1), C13(7)
4	RY-2	Ruyuan, Guangdong	113°11′02″, 25°00′40″	ssp. *obconica*	11	0.00030 (0.00012)	0.473 (0.162)	C9(1), C13(8), C15(2)
5	SZ-2	Sangzhi, Hunan	110°01′18″, 29°32′00″	ssp. *obconica*	9	0.00036 (0.00013)	0.556 (0.165)	C5(2), C6(1), C7(6)
6	YC-1	Yichang, Hubei	111°20′06″, 30°53′19″	ssp. *obconica*	6	0.00020 (0.00013)	0.333 (0.215)	C1(5), C2(1)
7	YC-2	Yichang, Hubei	110°59′04″, 30°58′00″	ssp. *obconica*	3	0	0	C8(3)
8	EMS	Emeishan, Sichuan	103°24′30″, 29°33′35″	ssp. *obconica*	6	0.00104 (0.00041)	0.600 (0.215)	C10(4), C11(1), C12(1)
	Lineage A: Yunnan group				27	0.00739 (0.00055)	0.855	
9	LQ	Luquan, Yunnan	102°28′34″, 25°34′09″	ssp. *begoniiformis*	5	0	0	C19(5)
10	DL	Dali, Yunnan	100°06′37″, 25°43′30″	ssp. *obconica*	5	0	0	C20(5)
11	WX	Weixi, Yunnan	99°15′48″, 27°11′15″	ssp. *obconica*	5	0	0	C18(5)
12	BS-1	Baoshan, Yunnan	99°06′49″, 25°16′42″	ssp. *obconica*	5	0.00048 (0.00028)	0.400 (0.237)	C16(4), C17(1)
13	BS-2	Baoshan, Yunnan	99°21′52″, 25°01′55″	ssp. *begoniiformis*	7	0.00017 (0.00012)	0.286 (0.196)	C21(6), C22(1)
	Lineage B: Sichuan group				28	0.00375 (0.00021)	0.804	
14	WWS	Wawushan, Sichuan	103°05′37″, 29°42′21″	ssp. *obconica*	5	0.00144 (0.00086)	0.400 (0.237)	C29 (1), C30(4)
15	LD	Luding, Sichuan	102°12′46″, 29°52′56″	ssp. *obconica*	5	0	0	C31 (5)
16	DJY-1	Dujiangyan, Sichuan	103°33′56″, 31°05′36″	ssp. *obconica*	5	0	0	C34(5)
17	DJY-2	Dujiangyan, Sichuan	103°34′43″, 31°08′40″	ssp. *obconica*	5	0	0	C34(5)
18	ML-1	Yanyuan-Muli,Sichuan	101°07′59″, 27°39′56″	ssp. *werringtonensis*	3	0	0	C33(3)
19	ML-2	Muli, Sichuan	101°16′50″, 27°56′31″	ssp.*werringtonensis*	5	0	0	C32(5)
	Lineage B: Central group				26	0.00164 (0.00012)	0.815	
20	SZ-1	Sangzhi, Hunan	109°59′57″, 29°33′54″	ssp. *obconica*	7	0.00017 (0.00012)	0.286 (0.196)	C23(6), C24(1)
21	NC	Nanchuan, Chongqing	107°12′55″, 29°02′40″	ssp. *obconica*	6	0	0	C25(6)
22	FJS	Fanjingshan, Guizhou	108°42′07″, 27°52′47″	ssp. *obconica*	6	0	0	C28(6)
23	XN	Xinning, Hunan	110°59′09″, 26°23′50″	ssp. *obconica*	7	0.00017 (0.00012)	0.286 (0.196)	C26(6), C27(1)

Sequence quality was checked against the original chromatogram and assembled using SeqMan™ (DNASTAR). For ITS sequencing, the presence of ‘double peaks’ at polymorphic sites for ITS in the chromatogram were check manually. Alleles (haplotypes) were first determined through haplotype subtraction [41]. Alternatively, orphaned alleles were resolved using PMD-18 vectors (TaKaRa) and by sequencing multiple clones. Resequencing was used to validate the occurrence of singletons for both the plastid DNA and ITS dataset. All haplotypes sequences (chlorotypes and ribotypes) were deposited in GenBank (Table S1).

### Nucleotide diversity and intraspesific divergence

Sequences for each fragment were aligned by Clustal X 1.18 [42], and further adjusted manually. Haplotype diversity (*H*_d_) and nucleotide diversity (π) were calculated using DnaSP 5.0 [43]. In addition, we also calculated within-population genetic diversity (*H*_S_), total diversity (*H*_T_), and two other parameters of population differentiation (*G*_ST_ and *N*_ST_) using PermutCpSSR 2.0 with 1000 permutations [44] (http://www.pierroton.inra.fr/genetics/labo/Software/PermutCpSSR/). *G*_ST_ is a parameter estimate based on haplotype frequencies; while *N*_ST_ takes into account the differences between haplotypes. The comparison of *G*_ST_ and *N*_ST_ was conducted by PermutCpSSR based on 1000 random permutations.

Analysis of molecular variance (AMOVA) was performed to assess the genetic differentiation among groups and between populations within groups (identified by phylogenetic analyses) using the program Arlequin 3.0 [45], and significance tests were conducted based on 1000 permutations.

### Phylogenetic reconstruction

Chlorotypes for the combined plastid fragments were generated by DnaSP 5.0 [43]. We performed Maximum Parsimony analysis (MP) and Bayesian Inference (BI) to infer the phylogenetic relationships among chlorotypes of *P. obconica*. The MP analysis was conducted by PAUP^*^ 4.0b10 with a heuristic search using the random addition of sequence method (1000 replicates) with the tree-bisection-reconnection (TBR) branch swapping, MULTREES, and BRANCHES COLLAPSED options selected [46]. The robustness of the trees was estimated by nonparametric bootstrapping (1000 replicates) [47]. The best-fitting model of GTR+G+I, calculated using Modeltest 3.7 based on Akaike's Information Criterion (AIC) [48], was applied to the BI analysis conducted by MrBayes 3.1.2 [49]. For each analysis, the Markov chain Monte Carlo (MCMC) algorithm was run for 2,000,000 generations with four simultaneous chains, starting from random trees and sampling one tree every 1000 generations. After the chains had become stationary, as judged from plots of likelihood and from split variances being <0.01, the first 10% of generations were discarded as burn-in. A majority rule consensus tree was constructed and posterior probabilities (PP) of nodes were calculated from the remaining samples. Based on the genus-wide phylogeny [50]–[51], *Primula barbicalyx* Wright, which, like *P. obconica*, belongs to the sect. *Obconicolisteri* Balf. f., and was used as an outgroup. In addition, a statistical parsimony network was constructed by TCS 1.13 [52]. Ambiguous connections (loops) in the networks were resolved following the procedure described by Crandall et al. [53].

### Nested Clade Analyses based on Chlorotype dataset

We defined hierarchic clades for Nested Clade Analysis (NCA) using the nested design rule [54]. The program GeoDis [55] calculates two main statistics on the nested cladogram: the clade distance (*D*_c_) and the nested clade distance (*D*_n_). These distances then were used to calculate an interior-tip statistic (I-*T*_c_ and I-*T*_n_) within each nested category based on the coalescent theory described by Posada et al. [56], as the interior distance minus the average tip distance. The significance of these statistics was estimated through a Monte Carlo procedure with 1000 random permutations [55]. The statistically significant results were then interpreted following the inference key (http://darwin.uvigo.es/software/geodis.html).

### Inferring demographic history

In order to test whether historical demographic expansion events have ever occurred in *P. obconica*, two neutrality tests, Tajima's *D* [57] and Fu's *Fs* [58] statistics, were calculated by Arlequin 3.0. Significantly negative Tajima's *D* indicates an excess of low-frequency alleles that can arise from purifying selection, rapid population expansion, and selective sweeps [59]. Fu's *Fs* is expected to have significantly large and negative values under conditions of demographic expansion [58]. The statistical significance of the estimates was calculated by Arlequin 3.0 with 1000 permutations.

Mismatch distribution analyses were used to examine demographic changes. The distributions of the frequency of observed and simulated pairwise differences among sequences were plotted by Arlequin 3.0 [60]. The shape of the observed mismatch distribution was tested against the null hypothesis of population expansion. The sum of squared deviations (*SSD*) between the observed and the expected mismatch distributions [61], and the raggedness index (*Rag*), were used to test the goodness-of-fit of the observation mismatch distribution to the expectation of a population expansion model [62]. If the sudden expansion model was not rejected, the relationship τ = 2μ*t* [60] was used to estimate the age of expansion (*t*), where μ is the substitution rate for DNA sequences.

Despite the uncertainty of the plant molecular clock in general and the lack of reliable fossil record for the genus *Primula*, a conventional molecular clock for the herb *trn*L-*trn*F region (8.24×10^−9^ subst. per site per yr, [63]) is employed in this study, as all species of *Primula* are perennial or annual plants that flower every year. In order to decide whether this rate is suitable for all plastid DNA combined, net average distance (*D*_a_) under Juke-cantor model was calculated for *trn*L-*trn*F and the combined data, respectively.

A Bayesian Skyline Plot (BSP), a method that does not rely on a prespecified parametric model of demographic history, was used to estimate past population dynamics over time from a sample of molecular sequences using a Markov chain Monte Carlo (MCMC) algorithm [64] with BEAST 1.4.7 [65]. We used this method to estimate changes in the effective population size (*N_e_*) of *P. obconica* since the time to the most recent common ancestor (TMRCA). We performed four MCMC runs for 10 million iterations, sampling genealogy and population size parameters every 1000 iterations and discarding the first 10% as burn-in. The linear growth model was selected for this analysis. We used an uncorrelated lognormal model [66] to account for rate variation among lineages with the nucleotide substitution model (GTR+G+I), though the mean substitution rate was fixed by an assumed molecular clock for herbs. Demographic history through time was reconstructed by the software Tracer 1.3 [67]. Time and effective population size were defined as million year and *θ* (*N_e_τ*; *τ*, generation time) for the BSP.

### Past and current distribution inference

In order to validate the impact of the length of cold periods (such as LGM) on the distribution of *P. obconica*, we inferred the distribution range using an Ecological Niche Model. Assuming the species has not changed its climatic preference, we reconstructed the range of *P. obconica* during the LGM according to its current distribution using a maximum entropy model (Maxent 3.1.0, [68]), which was considered to be more robust than other methods (cf. [69]). The current distribution information for *P. obconica* was estimated from collection records of the species from three main herbaria in China (IBSC, KUN and PE), and sampling sites in this study were also added to cover the whole distribution range (Table S2). Bioclimatic variables of current conditions and the LGM data at 2.5′ spatial resolution were downloaded from the worldClim database (http://www.worldclim.org/, [70]). We used LGM data simulated by the Model for Interdisciplinary Research on Climate (MIROC) [71]. In a preliminary investigation, we first chose all 19 environmental parameters to model the potential distribution of the species, but discarded several parameters from further analysis due to their low contribution levels (<1% as shown by their Maxent result). We chose 10 replicate runs in each analysis to ensure more reliable results. In order to test the performance of each model, 20% of the data in each run was randomly selected by Maxent and compared with the model output created with the remaining data. The area under the receiver operating characteristic curves (AUC) was used to compare model performance [68].

## Results

### Plastid DNA sequence variation and genetic structure

For the plastid DNA dataset, a total of 34 chlorotypes was identified when *trn*L-*trn*F and *rps*16 were combined, including 77 site substitutions and 6 indels. The geographical distribution of the chlorotypes was highly structured (see Figure 1 and Table 1). Populations were fixed with unique chlorotypes, except for populations RY-1, RY-2 and LC from Guangdong province and populations DJY-1 and DJY-2 from Sichuan province, which shared C13 and C34, respectively. Haplotype diversity (*H*_d_) varied among populations. High *H*_d_ values were found in central China (e.g. SZ-2) and southeastern China (e.g. RY-1 and RY-2), while populations from southwest China always exhibited relatively low haplotype diversity (Table 1). The mean nucleotide diversity (π) also varied among populations, though the value at the species level was relatively high (0.00827). Yunnan and Sichuan group, both in southwest China, had higher π values when compared with Central and Eastern groups (Table 1). Among populations in Southwest China, WWS and EMS, both located in the eastern edge of the Sichuan basin, had relatively high π values (0.00144 and 0.00104, respectively). By contrast, the π value in RY-1 (0.00119) was the highest against that of populations from Central and Eastern groups (Table 1).

The total genetic diversity across all populations (*H*_T_ = 0.988±0.0096) was larger than the average within-population diversity (*H*_S_ = 0.179±0.0435). High values of *G*_ST_ and *N*_ST_ (0.819 and 0.976, respectively) indicated that there is significant population differentiation in *P. obconica*. The species was confirmed to have significant phylogeographic structure as *N*_ST_ was significantly greater than *G*_ST_ (1000 permutations, *P*<0.05). Results from analyses of the molecular variance (AMOVA) among groups and between and within populations are shown in Table 2.

**Table 2:** Results of analyses of molecular variance (AMOVA) for two datasets for all populations and population groups of *Primula obconica*.

.Source of variation	plastid DNA data					ITS data				
	d.f.	SS	VC	PV (%)	Fixation index	d.f.	SS	VC	PV (%)	Fixation index
Lineage A (Eastern group vs. Yunnan group)					*F*_ST_ = 0.96526*					*F*_ST_ = 0.91535*
Among groups	1	139.419	3.28927	51.68	*F*_SC_ = 0.92810*	1	210.899	2.67706	38.53	*F*_SC_ = 0.86229*
Among populations within groups	11	206.426	2.8538	44.84	*F*_CT_ = 0.51684*	11	407.478	3.68261	53.00	*F*_CT_ = 0.38531*
Within populations	72	15.919	0.2211	3.47		119	69.987	0.58813	8.46	
Lineage B (Central group vs. Sichuan group)					*F*_ST_ = 0.96197*					*F*_ST_ = 0.82214*
Among groups	1	57.581	1.57357	36.00	*F*_SC_ = 0.94059*	1	118.769	1.92908	30.69	*F*_SC_ = 0.74338*
Among populations within groups	8	113.642	2.63175	60.20	*F*_CT_ = 0.35996*	7	225.768	3.23837	51.52	*F*_CT_ = 0.30692*
Within populations	44	7.314	0.16623	3.80		79	88.315	1.11792	17.79	
Lineage A vs. Lineage B					*F*_ST_ = 0.98142*					*F*_ST_ = 0.90355*
Among groups	1	455.921	6.51599	60.44	*F*_SC_ = 0.95304*	1	338.598	2.70269	32.61	*F*_SC_ = 0.85688*
Among populations within groups	21	517.068	4.06522	37.71	*F*_CT_ = 0.60437*	20	962.914	4.78684	57.75	*F*_CT_ = 0.32606*
Within populations	116	23.234	0.20029	1.86		198	158.302	0.79951	9.65	
Total populations					*F*_ST_ = 0.97335*					*F*_ST_ = 0.88502*
Among populations	22	972.989	7.31542	97.34		21	1301.511	6.15395	88.50	
Within populations	116	23.234	0.20029	2.66		198	158.302	0.79951	11.50	

d.f., degrees of freedom; SS, sum of squares; VC, variance component; *F*_CT_, correlation of haplotypes within groups relative to total; *F*_SC_, correlation within populations relative to groups; *F*_ST_, correlation within population relative to total;

‘*’ , *P* values<0.05.

### ITS sequence variation

Individuals fixed for unique chlorotypes were sequenced with ITS primer pairs. ITS sequences from BS-2 and DJY-2 were not included in further analyses due to failure in amplification and/or sequencing. In cloning analyses, a total of 5–10 ITS clones was sequenced for each sample. A total of 220 sequences across the 110 individuals was obtained from the 21 populations we examined. The alignment length was 692 bp with 110 nucleotide substitution sites without indels. Sixty-six ribotypes were retrieved from the ITS matrix. The ribotypes in each population and the corresponding nucleotide parameters are shown in Table 3. The mean nucleotide diversity (π) was 0.01935 (±0.00056), but it varied between populations (Table 3). Across all populations, SZ-2 from the Sangzhi region of central China had the highest nucleotide diversity (π = 0.0134). Populations from Southwest China, such as DJY-1, WWS, DL and BS-1, generally exhibited higher nucleotide diversity than that of populations from eastern and central China. The total genetic diversity across all populations (*H*_T_ = 0.994±0.0081) was larger than the average within-population diversity (*H*_S_ = 0.571±0.0558).

**Table 3:** The ribotypes detected from the ITS dataset and the corresponding genetic diversity.

Nos.	Populations codes	N	π (sd)	*H*_d_ (sd)	Ribotypes (nos. of individuals)
	Lineage A: Eastern group	43	0.00865 (0.00263)	0.852 (0.030)	
1	NP	6	0.00044 (0.00021)	0.303 (0.147)	R53(10),R54(2)
2	LC	7	0.00078 (0.00009)	0.538 (0.06)	R48(8), R51(6),
3	RY-1	8	0.00066 (0.00014)	0.458 (0.095)	R48(11),R52(5)
4	RY-2	8	0.00086 (0.00026)	0.525 (0.137)	R48(11), R49(2), R50(2), R52(1)
5	SZ-2 *	6	0.0134 (0.00151)	0.545 (0.062)	R18(6), R56(6)
6	YC-1	5	0.00170 (0.00037)	0.800 (0.100)	R42(1), R43(1), R44(1), R45(4), R55(3)
7	YC-2	3	0.00077 (0.00025)	0.533 (0.172)	R46(4), R47(2)
	Lineage A: Yunnan group	26	0.01727 (0.00521)	0.919 (0.017)	
8	ML-1	2	0	0	R57(4)
9	DL	3	0.00983 (0.00475)	0.867 (0.129)	R63(2), R64(1), R65(2), R66(1)
10	WX	5	0.00058 (0.00030)	0.378 (0.181)	R31(8), R32(1), R33(1)
11	BS-1	4	0.00924 (0.00336)	0.750 (0.139)	R1(4), R2(2), R61(1), R62(1)
12	LD	4	0.00983 (0.00475)	0.867 (0.129)	R58(4), R59(3), R60(1)
13	LQ	8	0.00077 (0.00007)	0.533 (0.046)	R34(8), R35(8)
	Lineage B: Sichuan group	20	0.01495 (0.00480)	0.921 (0.025)	
14	EMS	5	0.00029 (0.00022)	0.200 (0.154)	R20(9), R21(1)
15	WWS	6	0.01036 (0.00151)	0.848 (0.074)	R36(2), R37(1), R38(1), R39(3), R40(1), R41(4)
16	DJY-1	4	0.00991 (0.00297)	0.929 (0.084)	R22(1), R23(1), R27(2), R28(1), R29(1), R30(2)
17	ML-2	5	0.00148 (0.00024)	0.689 (0.104)	R24(5), R25(2), R26(3)
	Lineage B: Central group	21	0.00504 (0.00190)	0.887 (0.031)	
18	SZ-1	6	0.00024 (0.00019)	0.167 (0.134)	R3(11), R4(1)
19	NC	4	0.00320 (0.00057)	0.929 (0.084)	R8(2), R9(1), R10(1), R11(2), R12(1), R13(1)
20	XN	7	0.00152 (0.0037)	0.659 (0.123)	R14(1), R15(8),R16(3), R17(1), R19(1)
21	FJS	4	0.00160 (0.00027)	0.679 (0.122)	R5(4), R6(1), R7(3)

‘*’ indicates that population of SZ-2 consists of two different ribotypes, which belongs to Eastern group and Central group, respectively.

We also detected significantly larger *N*_ST_ values (0.844±0.0380) compared to *G*_ST_ (0.425±0.0558), showing there is phylogeographic structure across the distribution range of *P. obconica*. AMOVA indicated that most of the variation (85.87%) related to differences between populations, while 15.13% of the variation was due to differences within populations (Table 2).

### Phylogenetic reconstruction and phylogeographic structure for both datasets

**Figure 2:**
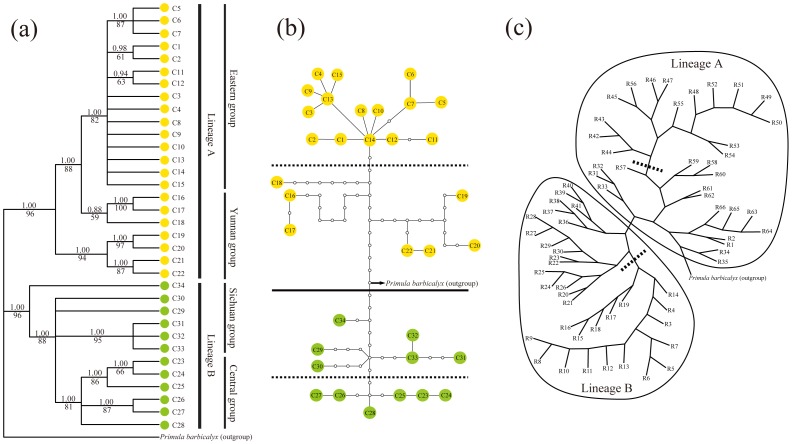
Phylogenetic relationships among haplotypes and lineage subdivergence detected in *Primula obconica*. (a) The phylogenetic topography based on plastid DNA dataset. Bootstrap values of Maximum Parsimony analysis and posterior probabilities of Bayesian inference are given above and below branches, respectively. (b) Maximum Parsimony networks of chlorotypes identified by TCS. Each solid line between circles represents one mutational step between two chlorotypes based on most parsimonious algorithm. The small open circles indicate the missing chlorotypes (not sampled or extinct). The solid line in the middle position of the network represents the two main lineages identified in the phylogenetic analysis, while the dashed line indicates the subdivision in each main lineage. The arrow indicated the connection between *Primua obconica* and *Primula barbicalyx*. Yellow and green circles in (a) and (b) correspond to lineage A and lineage B, respectively, as shown in Figure 1. (c) The strict consensus of the Maximum Parsimony trees of ribotypes. The two main lineages are circled by a solid line, while a dashed line in each circle represents the subdivision in each main lineage. The terminal of each branch represents haplotype recovered from plastid DNA and ITS datasets (See Table 1 and 3).

Highly concordant trees were produced from MP and BI analyses for the plastid DNA dataset (Figure 2a). Chlorotypes of *P. obconica* were separated into two major lineages (lineage A and lineage B) with strong supporting values (Figure 2a). Populations from Yunnan province and east China (corresponding to the Yunnan group and Eastern group, respectively) were included in lineage A, while lineage B comprised populations from the Sichuan province and central China (corresponding to the Sichuan group and Central group, respectively). It is noteworthy that chlorotypes (C10, C11 and C12) from population EMS in the Sichuan province were placed in lineage A (Figure 1, Figure 2a). The evolutionary relationships in lineage B were poorly resolved, but there was a well-supported clade (Central group) comprising populations from the Chongqing, Hunan and Guizhou provinces in central China. The haplotype network constructed by TCS also revealed the evolutionary relationships within and between several geographically-structured groups (Figure 2b), e.g. chlorotypes from Yunnan and Sichuan are considered to be older than those from central and east China, since they connect with outgroup directly and were located in an intermediate position [81] (Figure 2b).

We could not obtain a well resolved phylogeny for the ITS dataset, but the most parsimonious tree shown in Figure 2c is largely consistent with the results from the plastid DNA dataset, which was supported by the Maximum Parsimony networks identified by TCS (data not shown). Ribotypes from Sichuan and Yunnan provinces are located in a central position, and each was connected with ribotypes from central and east China. However, ITS ribotypes from the EMS population were linked with other ribotypes from the Sichuan province, which contrasts with the plastid DNA results. Other difference was also found in populations ML-1 and LD (Figure 2a, b, c). In addition, ribotypes R18 and R56 from population SZ-2 were grouped with the Central group and Eastern group, respectively.

### Historical demography of *Primula obconica*

**Figure 3:**
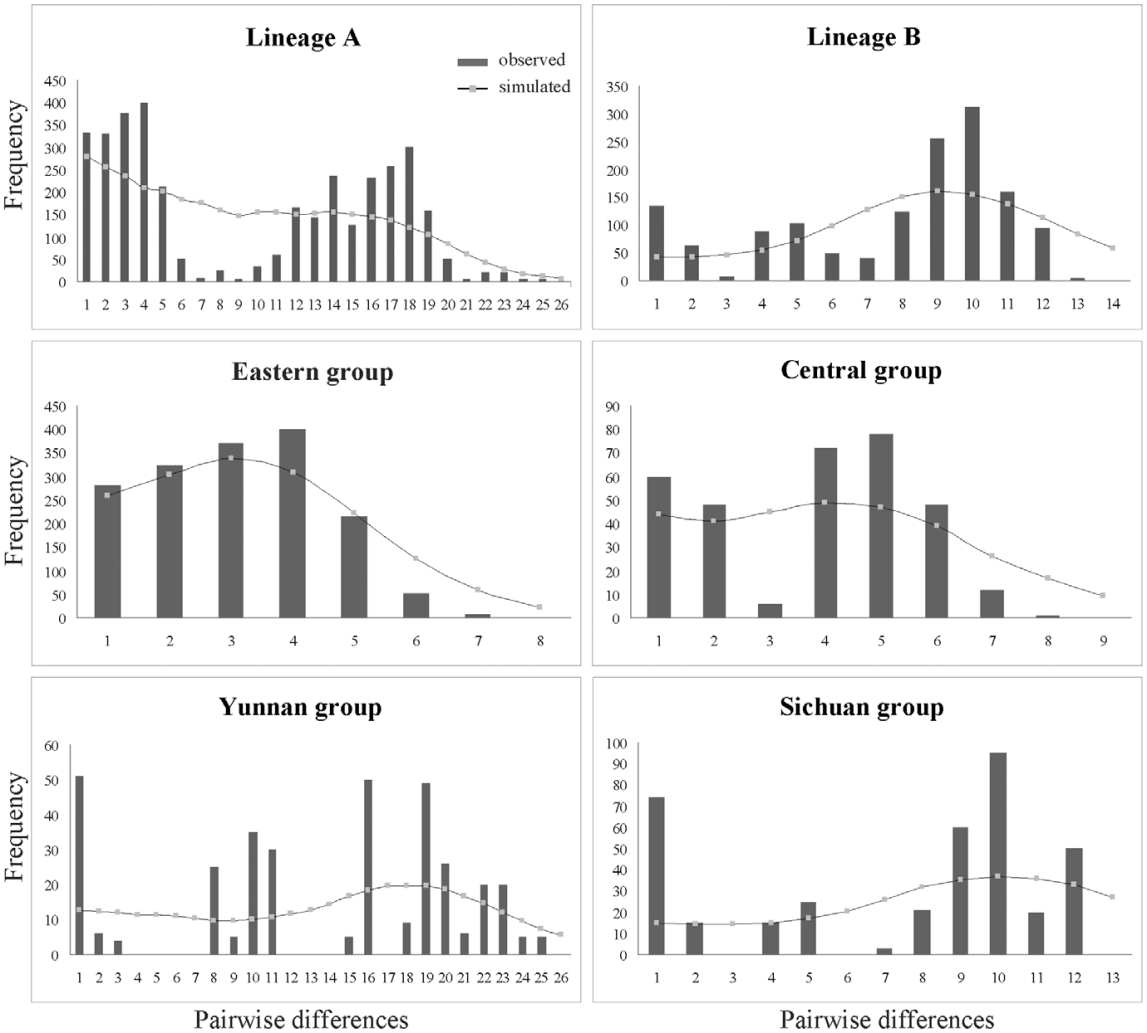
Mismatch distribution analysis of the plastid DNA dataset for lineage A, lineage B, the Eastern group, the Central group, the Sichuan group and the Yunnan group. Histograms correspond to observed frequencies of pairwise nucleotide differences, and lines represent the simulated frequencies under the sudden expansion model.

No significantly negative values for Tajima's *D* or positive values for Fu's *Fs* were detected at the species level (Table 4). However, when the two neutrality tests were examined for each of the six groups (lineage A, lineage B, Eastern group, Central group, Sichuan group, and Yunnan group), significantly negative results were only found in the Eastern group (Table 4). Similarly, the Eastern group generated a bell-shaped unimodal curve with no significant *SSD* and *Rag* values in Mismatch Analysis (Figure 3, Table 4).

**Table 4:** Results of historical demographic analyses and population genetics statistics based on two datasets for population groups.

	plastid DNA						ITS					
Groups	Population	Tajima's *D*	Fu's *Fs*	*SSD* (*P* value)	*Rag* (*P* value)	*τ* (95% CI)	Population combination	Tajima's *D*	Fu's *Fs*	*SSD* (*P* value)	*Rag* (*P* value)	*τ* (95% CI)
	Combination											
Lineage A		−0.873	−0.251	0.019 (0.518)	0.012 (0.787)	15.752 (6.171–28.289)		−0.412	−1.437	0.008 (0.856)	0.009 (0.764)	20.644 (9.533–34.751)
Eastern group	NP, YC-1, YC-2, SZ-2, RY-1, RY-2, LC, EMS	−1.751*	−6.186**	0.005 (0.425)	0.025 (0.75)	2.866 ( 1.117–5.028)	NP, YC-1, YC-2, SZ-2^a^, RY-1, RY-2, LC	−0.696	−3.446	0.005 (0.804)	0.016 (0.938)	4.192 (0.792–9.771)
Yunnan group	BS-1, BS-2, DL, LQ, WX	1.285	8.618	0.053 (0.004)	0.101 (0.003)	18.703 (9.061–36.143)	BS-1, DL, LQ, WX, ML-1, LD	0.788	3.527	0.033 (0.007)	0.04 (0.004)	20.242 (10–823–31.534)
Lineage B		0.332	2.221	0.031 (0.001)	0.04 (0.023)	8.764 (3.392–14.12)		−0.619	−5.585	0.004 (0.706)	0.011 (0.28)	12.561 (5.144–20.008)
Central group	NC, FJS, XN, SZ-1	−0.16	1.211	0.038 (0.113)	0.082 (0.198)	4.481 (1.078–11.539)	NC, FJS, XN, SZ-1, SZ-2^b^	0.285	−3.151	0.008 (0.616)	0.024 (0.518)	6.099 (1.108–14.471)
Sichuan group	DJY-1, DJY-2, LD, ML-1, ML-2, WWS	1.296	5.258	0.065 (0.004)	0.117 (0.014)	10.032 (3.888–26.086)	DJY-1, ML-2, WS, EMS	0.049	0.198	0.022 (0.023)	0.055 (0)	12.561 (5.592–18.498)
Total	All populations	−0.245	0.354	0.008 (0.435)	0.009 (0.180)	20.217 (9.073–32.812)	All populations	−0.853	−14.79	0.002 (0.797)	0.005 (0.490)	16.449 (8.655–23.096)

‘*’ , *P* values<0.05;

‘**’ , *P* values<0.01; superscript ‘a’, individuals fixed with ribotypes R56; superscript ‘b’, individuals fixed with R18; *SSD*, sum-of-squared deviations; *Rag*, Raggedness index.

Net average distance (*D_a_*) between Lineage A and Lineage B was estimated to be 0.008 when all plastid DNA fragments combined, approximately equal to that of *trn*L-*trn*F alone (0.0077). We then extended the assumed substitution rate of *trn*L-*trn*F to the combined plastid DNA data. We used the expression *τ* = 2μ*t* to estimate the expansion ages of the Eastern group. Based on the aligned sequence length (1700 bp) and a estimated generation time of one year, the expansion of the Eastern group might occur at ca. 0.104 Myr (95% CI: 0.0495–0.169 Myr).

**Figure 4:**
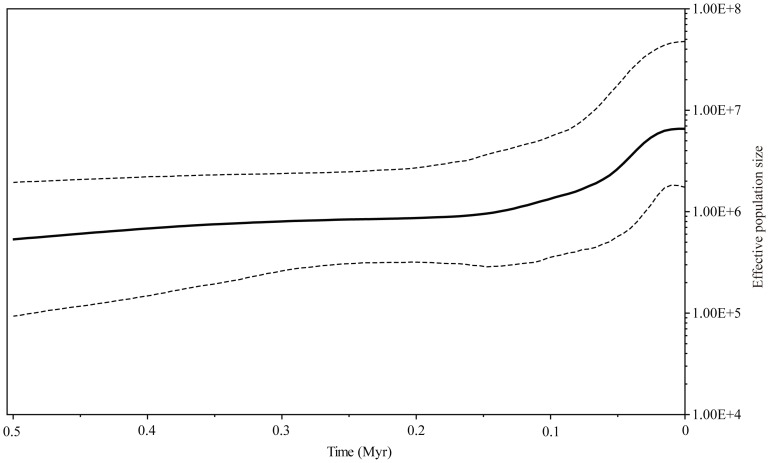
Bayesian Skyline Plot representing the historical demographic trend in plastid lineages of *Primula obconica*. The x-axis units are years before present and was estimated based on a substitution rate of 8.24×10^−9^ subst. per site per yr. The y-axis shows an estimate of effective population size (*N*_e_ *τ*), the product of effective population size and generation length in million years (Myr). The bold black line represents the median estimates through time from the Bayesian posterior distribution, while the dashed line shows the 95% highest posterior density (HPD) boundaries.

Figure 4 shows a Bayesian Skyline Plot (BSP) in which the demographic history of *P. obconica* is illustrated in terms of effective population size. Prior to ca. 0.1 Myr, the effective population size seems to have been relatively stable or to have increased only gradually. Subsequently, there was a substantial growth period followed by another period of relative stability that has persisted since the onset of the Holocene.

### Nested Clade Analysis results

The most parsimonious network constructed by TCS contained two ambiguous connections (loops), which were resolved using the procedure described by Crandall et al. [53]. The nested cladogram contained five hierarchical levels with 14 polymorphic clades (Figure S1, Table S3). A nested clade analysis using 1000 permutations was applied to these clades and did not reject the null hypothesis of no association with geographical location (*P*<0.05), except that Clade 1-1 contained chlorotypes (C3, C4, C9, C13, and C15) from the northern area of Guangdong (Nanling Mountain). According to the NCA analysis, Clade 1-1 evolved as a result of restricted gene flow due to isolation by distance, while most of the remaining clades probably resulted from previous gradual range expansions followed by fragmentation or contiguous range expansion (Table S3).

### Inferring distribution patterns with Ecological Niche Modeling

**Figure 5:**
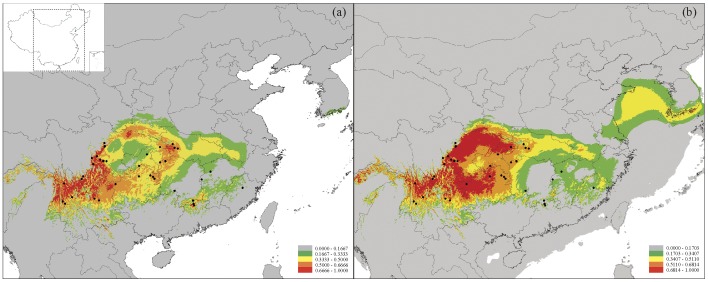
Inferred distribution range of *Primula obconica* in subtropical China simulated by an Ecological Niche Model (ENM) using bioclimatic variables. (a) The current potential distribution and (b) the simulated distribution range at the Last Glacial Maximum (LGM). Marginal to optimal habitat are shown in green (marginal) to red (optimal). Black circles represent collecting records included in our analysis.

The inferred current and past (LGM) distribution of *Primula obconica* is shown in Figure 5. The AUC values based on both training and test presence data for the current and LGM periods were all higher than expected by chance (0.997 and 0.993, 0.996 and 0.993, respectively), which demonstrates good model performance. Compared with the two simulated distributions, it is remarkable that most suitable habitats for *P. obconica* occur in southwest and central China. In addition, the inferred distribution during the LGM is slightly larger than the current distribution; for example, the areas of the Sichuan basin and the eastern plain of China that were suitable for the species were both slightly larger during the LGM (Figure 5).

## Discussion

### Genetic differentiation between populations of *Primula obconica*

*Primula obconica* exhibited high genetic differentiation between populations, but extremely low genetic diversity within populations (Table 2). The average genetic differentiation in angiosperms, determined using maternally inherited markers, is 0.67 [72], but the value obtained for *P. obconica* is much greater than this. Plastid genomes in most angiosperm species are maternally inherited [73]. This is the case in two other primrose species [74], and it is likely true for *P. obconica* as well. Gene flow estimations using plastid DNA markers are based on DNA transmission through seeds. However, all *Primula* species lack efficient seed dispersal mechanisms; their seeds are ejected from the fruit capsules when they ripen and are dispersed by gravity or occasionally as a result of transport in flood waters, though seed dispersal by ants or rodents occasionally occurs in some *Primula* species [75]. According to our field observations, seeds of *P. obconica* are mainly dispersed by gravity, which may be one of the main reasons for the high levels of genetic differentiation between populations.

In addition, environmental conditions in mountains at higher elevations differ markedly from those in the intervening valleys, which probably act as barriers to gene flow, and further result in high levels of inter-population genetic divergence [76]–[77]. Subtropical China is famous for its mosaic of mountains, which isolated populations of montane species in patch-like habitats. This is the case in most studied species of subtropical China, such as *Eurycorymbus cavaleriei* [27], *Dysosma versipellis* [78], *Dipentodon longipedicellatus* [79], and *Taxus wallichiana* [23]. As a montane herb, *Primula obconica* is widely distributed across major mountain ranges of subtropical China (Figure 1) [14], [29]. The species always occurs in shaded wet areas in thickets or forests with a range of varied elevation from 500–3300 m a.s.l.. Considering the fact of the mosaic of mountains and cultivated land area in low-elevation mountains, the mixed evergreen and deciduous broadleaved forests in subtropical China are fragmented, which lead to patch-like habitats of *P. obconica*. In the present study, the result that the majority of the populations were fixed with unique haplotypes suggests that there is limited gene flow between populations. Although there is some variation in the morphological traits of *P. obconica*, we did not find any correlation between haplotypes and subspecies since all haplotypes were mixed with each other in both plastid DNA and ITS datasets (Figure 2a, b, c). Adaptation to local environments may have resulted in genetic and morphological differentiation, but the populations have not had enough time to sort their lineages out (incomplete lineage sorting).

### The area of origin and underlying migration events

Based on extensive data for the genus *Primula*, Hu [80] suggested that southwest China is the biodiversity and distribution centre for this genus, although whether the region acted as the origin area or a refugium in cold periods remains unknown. In theory, older haplotypes have a greater probability of becoming interior haplotypes than younger haplotypes in a network [81]. In the present study, the relatively older haplotypes along with the increased divergence of haplotypes in southwest China (Yunnan and West Sichuan) clearly indicate that *P. obconica* originated there (Figure 2b).

Since theory predicts that genetic drift should be reduced in an exponentially growing population, and that the replacement of ancestral haplotypes is enhanced at the leading edge of an expanding population [25], we were able to infer the likely expansion direction from the positions of specific haplotypes. The fact that older haplotypes were all found in southwest China indicated that there were two eastward migration routes (Yunnan to east China, and Sichuan to central China). This result strongly supports the hypothesis of eastward migration routes in subtropical China proposed by Wang [82]–[83]. Wang hypothesized that many plants originated from southwest China and migrated to the east along several mountain ranges, such as Qin-Daba Mountains in the north, the Dalou and the Wuling Mountains in central China, and the Nanling Mountains in south China [82]–[83] (Figure 1). We did not identify any migration fingerprints along mountains and/or river systems flowing east or west. However, the Nanling Mountains might actually be a barrier rather than a corridor for *P. obconica*, since the mountain populations only contained newly evolved chlorotypes (such as Clade 2-1, Table S3). NCA results also confirmed the occurrence of migration events (Table S3).

It is noteworthy that many chlorotypes of lineage A are missing in the geographic gap between Yunnan and east China (with the exception of the chlorotypes of the EMS population) (Figure 1, Figure 2a, b). During the Holocene, the greatest effective precipitation caused by the East Asian and Indian monsoons resulted in large and well-developed Karst areas in the southeast Yungui plateau (Guizhou and Guangxi Provinces) [84]. Moreover, severe rocky desertification in the region due to anthropogenic activity might increase the likelihood of losing key haplotypes linking the Yunnan and east China lineages [85].

### The expansion model of *Primula obconica*

Given the highly complex topography in Subtropical China [13] and the limited impact of glaciation during the Pleistocene [21], [86], it is less likely to find population expansion patterns that are best described by the pioneer model [2]. The preference for montane habitats of *P. obconica* might result in the limited dispersal distance that allowed constant subsequent dispersal in a manner of the phalanx model [6]–[7]. In this study, gradual expansion events were frequently identified in many clades (Figure S1; Table S3), and a gradually increasing or stable effective population size was indicated by the BSP analysis (Figure 4). Results from Mismatch Analysis and neutrality test also supported its relatively stable population size (Table 4). As shown in the phalanx model, the gradual population expansion in this species might reflect a slower expansion rate from refugia, as the warm and moist environmental conditions in the interglacial periods caused the species to retreat up into higher elevations forming isolated patch-like populations [7].

The phalanx model for *P. obconica* was further validated by modeling the distribution of the species, as it had a slightly larger distribution range in cold periods (i.e. during the LGM) than in warm periods (the present-day distribution) (Figure 5). Range expansion in this subtropical species following the phalanx model is likely to support the hypothesis of multiple refugia within the subtropical region [23]–[27], but stands in contrast to the traditional idea that there were three main refugia across the region based on their high level of endemism [15].

Although the phalanx model probably explains the general gradual range expansions of *P. obconica*, different environments will have affected the historical demography of the species. For example, we found that the Eastern group has been expanding rapidly on the eastern plain of China approximately since the last 0.104 Myr (Figure 3, Table 4). This region contains many plains and hills with elevations below 1000 m a.s.l. These low altitudes are likely to have played an important role in the demography of *P. obconica* across its range (Figure 5, Table 4), where individuals migrated down from hills, and spread across the plains in cold periods. Unfortunately, despite the rapid expansion simulated in our study, there are relatively few populations remaining in eastern China. The limited and scattered current distribution of *P. obconica* is likely due to climatic warming since the Holocene and overexploitation by humans. In addition to the historical demography of *P. obconica*, more genetic differentiations have been identified in different environments, which are discussed below.

### The effects of environmental heterogeneity on lineage divergence

East Asia can be divided into three geographical zones, with a pronounced decrease in altitude from west to east [13] (See inset map in Figture 1). The lineage differentiation in *P. obconica* coincides almost perfectly with this geographic division. For example, the lineage split of Lineage B (Sichuan and Central groups) is consistent with the division between the HHM and the Yungui Plateau (Figure 1).

In this study, a more significant lineage division (Central group and Eastern group) occurred in the transitional belt between the Yungui Plateau and the eastern plain (Figure 1). In particular, chlorotypes SZ-1 and SZ-2, which were located at different elevations in the Taiping Mountain, belonged to Eastern group and Central group, respectively. The occurrence of lineage divergence between the two regions was further supported by the ITS data. Furthermore, two ribotypes (R18 and R56) in population SZ-2 belonged to different lineages (Figure 2c), respectively, and caused the highest nucleotide diversity in this population (Table 3). The pattern of different coexisting lineages in the region is similar to the category II intraspecific pattern suggested by Avise [87], which means the suture line is a secondary admixture zone between allopatrically evolved populations [87]. Following the interpretation of Mismatch Analysis results of Pauls et al. [88], we were able to detect expansion signal in Eastern group (Figure 3, Table 4), although the Central group might have experienced a gradual expansion (Table S3, see Clade 3–9).

Another interesting division occurred in the HHM. Recent surveys of flora in this region have shown a major change in species composition across the 29°N latitudinal line in the HHM, dividing it into southern and northern sub-regions [89]–[90]. There are a series of high mountains and plateaus along the 29°N latitudinal line, including Mt. Gongga and 28 other surrounding mountains in the east, Meili Snow Mountain and the Mangkang Plateau in the west, and the Litang-Daocheng Plateau in the center [91]–[92]. Especially, the fact that many peaks in excess of 5500 m a.s.l. in the Litang-Daocheng Plateau, probably resulted in the earliest and largest glaciation during the Pleistocene in the HHM (so-called Daocheng glaciation, [21]). The special topography across the latitudinal line may result in the major difference in flora in the HHM, since these mountains and plateaus form a significant barrier for the dispersal of species across the region [90]. In the current study, two ancestral lineages of *P. obconica* (the Yunnan and Sichuan lineages) are located in the HHM, which split geographically with the 29°N latitudinal line. Similar genetic patterns were also found in other alpine plants in this area, such as *Primula secundiflora* [93] and *Ligularia tongolensis* [94]. Our results, based on intraspecific genetic data, support the hypothesis of a 29°N latitudinal geographic barrier, but warrants further evidences.

### Conclusion

In this study, we identified two main lineages in *P. obconica*, each comprising two subclades. We found that the species originated from Southwest China and then migrated to eastern and central China, since the relatively older lineages occurred in Southwest China. We also detected that *P. obconica* populations experienced a gradual expansion and had relatively larger distribution range during LGM than at present, which firstly provided the evidence to support the hypothesis of the phalanx model as a main expansion manner for montane species in subtropical China. Finally, we found that lineage divergence was highly correlated with many geographical divisions, highlighting the importance of environmental heterogeneity in maintaining the present-day lineage differentiation in subtropical China.

## Supporting Information

Figure S1 The nested cladogram of chlorotypes of *Primula obconica*.
(DOC)

Table S1 GenBank accession numbers identified in this study.
(DOC)

Table S2 The distribution information of *Primula obconica* examined in this study.
(DOC)

Table S3 Nested contingency analysis of geographical structure based on 1000 permutation and chain of inference based on GeoDis inference key.
(DOC)
