# Supplementary material for: Population Expanding with the Phalanx Model and Lineages Split by Environmental Heterogeneity: A Case Study of Primula obconica in Subtropical China
Source: PLoS One. 2012 Sep 19;7(9):e41315. doi: 10.1371/journal.pone.0041315 (PMC3446961; doi:10.1371/journal.pone.0041315)
Supplement: Figure S1 — The nested cladogram of chlorotypes of Primula obconica. (DOC) [file pone.0041315.s001.doc]

Figure S1 The nested cladogram of chlorotypes of *Primula obconica*.
