# Supplementary material for: Population Expanding with the Phalanx Model and Lineages Split by Environmental Heterogeneity: A Case Study of Primula obconica in Subtropical China
Source: PLoS One. 2012 Sep 19;7(9):e41315. doi: 10.1371/journal.pone.0041315 (PMC3446961; doi:10.1371/journal.pone.0041315)
Supplement: Table S1 — GenBank accession numbers identified in this study. (DOC) [file pone.0041315.s002.doc]

Table S1 Nested contingency analysis of geographical structure based on 1000 permutation and chain of inference based on GeoDis inference key.

| Clade | Subclade / haplotype | Permutational chi-square statistic (χ^2^) | Probability | Inference chain | Inference |
| --- | --- | --- | --- | --- | --- |
| Clade 1-1 | C3, HC4, C9, C15, C13 | 11.4883 | 0.0950 | 1-2-3-4-NO | Restricted gene flow with isolation by distance |
| Clade 1-3 | C8, C10, C12, C14 | 28.0000 | 0.0000 | 1-2-11-12-13-21-NO | Past gradual range expansion followed by fragmentation |
| Clade 1-15 | C32, C33 | 8.0000 | 0.0120 | 1-19-20-NO | Inadequate geographical sampling |
| Clade 2-1 | 1-1, 1-2, 1-3, 1-4, 1-5 | 180.2143 | 0.0000 | 1-2-11-12-13-21-NO | Past gradual range expansion followed by fragmentation |
| Clade 2-12 | 1-17, 1-18 | 13.0000 | 0.0000 | 1-19-20-2-3-5-6-13-21-NO | Past gradual range expansion followed by fragmentation |
| Clade 3-3 | 2-4, 2-5 | 10.0000 | 0.0060 | 1-19-20-NO | Inadequate geographical sampling |
| Clade 3-7 | 2-8, 2-9 | 13.0000 | 0.0010 | 1-19-20-NO | Inadequate geographical sampling |
| Clade 3-9 | 1-19, 2-12, 2-13 | 52.0000 | 0.0000 | 1-2-11-12-13-21-NO | Past gradual range expansion followed by fragmentation |
| Clade 4-1 | 3-1, 3-2 | 45.8756 | 0.0000 | 1-19-20-2-11-17-NO | Inconclusive outcome |
| Clade 4-2 | 3-3, 3-4, 3-5 | 44.0000 | 0.0000 | 1-19-20-NO | Inadequate geographical sampling |
| Clade 4-4 | 3-7, 3-8, 3-9 | 88.0000 | 0.0000 | 1-19-20-2-11-12-NO | Contiguous range expansion |
| Clade 5-1 | 4-1, 4-2 | 83.0000 | 0.0000 | 1-19-20-2-11-12-13-21-NO | Past gradual range expansion followed by fragmentation |
| Clade 5-2 | 4-3，4-4 | 54.0000 | 0.0000 | 1-19-20-NO | Inadequate geographical sampling |
| Total cladogram | 5-1,5-2 | 139.0000 | 0.0000 | 1-2-11-12-NO | Contiguous range expansion |
