# Supplementary material for: Population Expanding with the Phalanx Model and Lineages Split by Environmental Heterogeneity: A Case Study of Primula obconica in Subtropical China
Source: PLoS One. 2012 Sep 19;7(9):e41315. doi: 10.1371/journal.pone.0041315 (PMC3446961; doi:10.1371/journal.pone.0041315)
Supplement: Table S2 — The distribution information of Primula obconica examined in this study. (DOC) [file pone.0041315.s003.doc]

Table S2 GenBank accession numbers identified in this study.

| Ribotypes | Accession # | Chlorotypes | Accession # | |
| --- | --- | --- | --- | --- |
|  | ITS |  | trnL-trnF | rps16 |
| R1 | JX258226 | C1 | JX258192 | JX258158 |
| R2 | JX258227 | C2 | JX258193 | JX258159 |
| R3 | JX258228 | C3 | JX258194 | JX258160 |
| R4 | JX258229 | C4 | JX258195 | JX258161 |
| R5 | JX258230 | C5 | JX258196 | JX258162 |
| R6 | JX258231 | C6 | JX258197 | JX258163 |
| R7 | JX258232 | C7 | JX258198 | JX258164 |
| R8 | JX258233 | C8 | JX258199 | JX258165 |
| R9 | JX258234 | C9 | JX258200 | JX258166 |
| R10 | JX258235 | C10 | JX258201 | JX258167 |
| R11 | JX258236 | C11 | JX258202 | JX258168 |
| R12 | JX258237 | C12 | JX258203 | JX258169 |
| R13 | JX258238 | C13 | JX258204 | JX258170 |
| R14 | JX258239 | C14 | JX258205 | JX258171 |
| R15 | JX258240 | C15 | JX258206 | JX258172 |
| R16 | JX258241 | C16 | JX258207 | JX258173 |
| R17 | JX258242 | C17 | JX258208 | JX258174 |
| R18 | JX258243 | C18 | JX258209 | JX258175 |
| R19 | JX258244 | C19 | JX258210 | JX258176 |
| R20 | JX258245 | C20 | JX258211 | JX258177 |
| R21 | JX258246 | C21 | JX258212 | JX258178 |
| R22 | JX258247 | C22 | JX258213 | JX258179 |
| R23 | JX258248 | C23 | JX258214 | JX258180 |
| R24 | JX258249 | C24 | JX258215 | JX258181 |
| R25 | JX258250 | C25 | JX258216 | JX258182 |
| R26 | JX258251 | C26 | JX258217 | JX258183 |
| R27 | JX258252 | C27 | JX258218 | JX258184 |
| R28 | JX258253 | C28 | JX258219 | JX258185 |
| R29 | JX258254 | C29 | JX258220 | JX258186 |
| R30 | JX258255 | C30 | JX258221 | JX258187 |
| R31 | JX258256 | C31 | JX258222 | JX258188 |
| R32 | JX258257 | C32 | JX258223 | JX258189 |
| R33 | JX258258 | C33 | JX258224 | JX258190 |
| R34 | JX258259 | C34 | JX258225 | JX258191 |
| R35 | JX258260 | *Primula barbicalyx* | FJ794251 | FJ786619 |
| R36 | JX258261 |  |  |  |
| R37 | JX258262 |  |  |  |
| R38 | JX258263 |  |  |  |
| R39 | JX258264 |  |  |  |
| R40 | JX258265 |  |  |  |
| R41 | JX258266 |  |  |  |
| R42 | JX258267 |  |  |  |
| R43 | JX258268 |  |  |  |
| R44 | JX258269 |  |  |  |
| R45 | JX258270 |  |  |  |
| R46 | JX258271 |  |  |  |
| R47 | JX258272 |  |  |  |
| R48 | JX258273 |  |  |  |
| R49 | JX258274 |  |  |  |
| R50 | JX258275 |  |  |  |
| R51 | JX258276 |  |  |  |
| R52 | JX258277 |  |  |  |
| R53 | JX258278 |  |  |  |
| R54 | JX258279 |  |  |  |
| R55 | JX258280 |  |  |  |
| R56 | JX258281 |  |  |  |
| R57 | JX258282 |  |  |  |
| R58 | JX258283 |  |  |  |
| R59 | JX258284 |  |  |  |
| R60 | JX258285 |  |  |  |
| R61 | JX258286 |  |  |  |
| R62 | JX258287 |  |  |  |
| R63 | JX258288 |  |  |  |
| R64 | JX258289 |  |  |  |
| R65 | JX258290 |  |  |  |
| R66 | JX258291 |  |  |  |
| *Primula barbicalyx* | JX258292 |  |  |  |
