# Supplementary material for: Population Expanding with the Phalanx Model and Lineages Split by Environmental Heterogeneity: A Case Study of Primula obconica in Subtropical China
Source: PLoS One. 2012 Sep 19;7(9):e41315. doi: 10.1371/journal.pone.0041315 (PMC3446961; doi:10.1371/journal.pone.0041315)
Supplement: Table S3 — Nested contingency analysis of geographical structure based on 1000 permutation and chain of inference based on GeoDis inference key. (DOC) [file pone.0041315.s004.doc]

Table S3 The distribution information of *Primula obconica* examined in this study.

| Vouchers | Locations | Longitude (E) | Latitude (N) |
| --- | --- | --- | --- |
| Wang s.n. | Nanping, Fujian, China | 118°12'48'' | 26°43'18'' |
| Xiong & Liu 4226 | Yifeng, Jiangxi, China | 114°48'00'' | 28°25'59'' |
| Xiong 4226 | Tonggu, Jiangxi, China | 113°51'52'' | 27°36'39'' |
| Ye s.n. | Lechang, Guangdong, China | 113°00'17'' | 25°01'00'' |
| Hao 392 | Ruyang, Guangdong, China | 113°03'42'' | 24°51'19'' |
| Hao 295 | Yichang, Hubei, China | 111°20'06'' | 30°53'19'' |
| Wang s.n. | Yichang, Hubei, China | 110°59'04'' | 30°58'00'' |
| Li 958 | Xingshan, Hubei, China | 110°47'18'' | 31°21'16'' |
| Liu 1772 | Longshan, Hunan, China | 109°27'21'' | 29°25'58'' |
| Chen 3589 | Yizhang, Hunan, China | 112°56'25'' | 25°22'14'' |
| Yan & Xu 001 | Xinning, Hunan, China | 110°59'09'' | 26°23'50'' |
| Tian 17987 | Sangzhi, Hunan, China | 110°01'18'' | 29°32'00'' |
| Hao 297 | Sangzhi, Hunan, China | 109°59'57'' | 29°33'54'' |
| Yan & Xu 004 | Nanchuan, Chongqing, China | 107°12'55'' | 29°02'40'' |
| Zhou & Su 107605 | Fengjie, Chongqing, China | 109°26'34'' | 31°02''40'' |
| Li s.n. | Fanjingshan, Guizhou, China | 108°42'07'' | 27°52'47'' |
| Zhang et al. 401623 | Yinjiang, Guizhou, China | 108°25'03'' | 28°02'02'' |
| Zhong 447 | Suiyang, Guizhou, China | 107°12'19'' | 27°56'20'' |
| Jiang 543 | Hezhang, Guizhou, China | 104°43'14'' | 27°07'29'' |
| Zhuang et al. 400370 | Jiangkou, Guizhou, China | 108°51'00'' | 27°41''19'' |
| Hu 8802 | Wenchuan, Sichuan, China | 103°32'04'' | 31°28''43'' |
| Luo 1712 | Zhongxie, Sichuan, China | 108°03'23'' | 30°15'56'' |
| Hao 432 | Emeishan, Sichuan, China | 103°24'30'' | 29°33'35'' |
| Jiang 33851 | Tianquan, Sichuan, China | 102°45'07'' | 30°04'00'' |
| Yang 57134 | Eshan, Sichuan, China | 103°27'06'' | 29°34'19'' |
| Xie 39573 | Shimian, Sichuan, China | 102°21'34'' | 29°13'38'' |
| Guang 6449 | Leshan, Sichuan, China | 103°45'47'' | 29°33'15'' |
| Hao 703 | Wawushan, Sichuan, China | 103°05'37'' | 29°42'21'' |
| Hao 448 | Luding, Sichuan, China | 102°12'46'' | 29°52'56'' |
| Hao 422 | Dujiangyan, Sichuan, China | 103°33'56'' | 31°05'36'' |
| Hao 443 | Dujiangyan, Sichuan, China | 103°34'43'' | 31°08'40'' |
| Hao 540 | Yanyuan-Muli,Sichuan, China | 101°07'59'' | 27°39'56'' |
| Hao 565 | Muli, Sichuan, China | 101°16'50'' | 27°56'31'' |
| Hao 378 | Luquan, Yunnan, China | 102°28'34'' | 25°34'09'' |
| Hao 524 | Dali, Yunnan, China | 100°06'37'' | 25°43'30'' |
| Hao 516 | Weixi, Yunnan, China | 99°15'48'' | 27°11'15'' |
| Hao & Yan 580 | Baoshan, Yunnan, China | 99°06'49'' | 25°16'42 |
| Hao & Yan 584 | Baoshan, Yunnan, China | 99°21'52'' | 25°01'55'' |
| Qiu 54219 | Songshan, Yunnan, China | 102°57''45'' | 25°23'10'' |
